# Hospital-based vaccination of older adult inpatients: comparison of two delivery models for influenza, COVID-19 and pneumococcal vaccines

**DOI:** 10.1186/s12879-026-12970-y

**Published:** 2026-03-02

**Authors:** Maria Lean, Garth Lean, Jeffrey J. Post

**Affiliations:** 1 Prince of Wales Hospital, Sydney, Australia; 2 Nuffield Department of Surgical Sciences, University of Oxford, Oxford, UK; 3 Translational Health Research Institute, Western Sydney University, Sydney, Australia; 4 School of Social Sciences, Western Sydney University, Sydney, Australia; 5 School of Clinical Medicine, The University of New South Wales, Sydney, Australia

**Keywords:** Vaccination uptake, Adult immunisation, Opportunistic vaccination, Hospital quality improvement, Health services research, SARS-CoV-2, Influenza, Pneumococcus

## Abstract

**Background:**

Vaccination coverage for influenza, SARS-CoV-2 and pneumococcal disease among older Australians remains suboptimal. Hospital admission is an underutilised opportunity for the vaccination of older adults.

**Methods:**

We conducted a 12-week prospective, single-centre evaluation at a tertiary hospital in Australia (August–November 2024). Inpatients aged ≥ 65 years admitted under cardiology, geriatrics, geriatric rehabilitation, respiratory and infectious diseases were screened for eligibility for National Immunisation Program (NIP)-recommended vaccines (influenza, SARS-CoV-2, conjugate pneumococcal). Stage 1 (weeks 1–6) involved academic detailing of medical officers (including education on the clinical benefit and standard of care for older adult immunisation) and weekly reminders to vaccinate as a part of routine care. During Stage 2 (weeks 7–12), we stopped giving reminders to medical officers, and a part-time vaccine prescriber (5 h/week) screened, consented and prescribed vaccines. Barriers to vaccination were recorded during both stages. We compared vaccination uptake between Stage 1 and Stage 2.

**Results:**

Of the 851 inpatients screened, 649 were eligible for 1,410 vaccines (76.3%), and 73 vaccinations were administered (5.2%). Overall uptake increased from 19/710 (2.7%) vaccinations during Stage 1, to 54/700 (7.7%) vaccinations during Stage 2 (OR 3.04, 95% CI 1.75–5.49; *p* < 0.001). An increase in vaccination uptake was observed for SARS-CoV-2 (Stage 1: 1/286, 0.3%; Stage 2: 12/280, 4.3%; OR 12.7, 95% CI 1.86–546.03) and pneumococcal vaccination (Stage 1: 8/247, 3.2%; Stage 2: 30/253, 11.9%; OR 4.0, 95% CI 1.75–10.34), but not for influenza (Stage 1: 10/177, 5.6%; Stage 2: 12/167, 7.2%; OR 1.29, 95% CI 0.5–3.44).

**Conclusions:**

Academic detailing and regular reminders for medical officers to vaccinate was associated with lower vaccination uptake of older adult inpatients than the use of a part-time vaccine prescriber.

**Supplementary Information:**

The online version contains supplementary material available at 10.1186/s12879-026-12970-y.

## Introduction

Increasing vaccination coverage among older adults is a public health priority. Older adults are highly susceptible to severe outcomes from infection due to comorbidities and age-related immune decline [1, 2]. Respiratory infections such as influenza, COVID-19, and pneumococcal disease pose particularly high risks. For these diseases, vaccination is one of the most effective interventions to reduce hospitalisation and mortality [3, 4]. For example, recent data show that updated SARS-CoV-2 booster doses in adults aged ≥ 65 years reduce COVID-19 mortality risk by up to 75% compared with adults vaccinated more than 12 months earlier [5].

In Australia, all adults aged ≥ 65 years are eligible for publicly funded influenza, SARS-CoV-2 and pneumococcal vaccines under the National Immunisation Program (NIP) [6], however, uptake remains suboptimal. According to the 2024 Annual Australian Respiratory Surveillance Report, only 61.1% of adults aged ≥ 65 years received an influenza vaccine [7]. Booster uptake for SARS-CoV-2 is also low, with an uptake of only 20.6% in adults ≥ 75 years, and 10.3% in adults aged 65–74 years as of April 2025 [8]. Coverage of the 13-valent pneumococcal conjugate vaccine among adults aged ≥ 70 years in 2023 was just 34.6% [9]. These gaps highlight the need for alternative vaccination strategies.

While primary care is the most common location for routine vaccinations, other vaccination settings such as nursing homes, outpatient clinics, and hospitals have been explored [10]. Admission to hospital, in particular, provides an important opportunity to vaccinate high-risk patients who may not routinely access primary care. Studies have shown that opportunistic inpatient vaccination is acceptable to staff, patients, and carers [11, 12], and several approaches to increase inpatient vaccination have been investigated, including computer-based reminder systems, staff education, standing-order protocols and nurse-immuniser models [13–16]. However, there is limited evidence about which delivery model is most effective at increasing vaccine uptake, especially in older adults.

This paper describes a pragmatic, prospective, single-centre evaluation that investigated two models of opportunistic vaccination of older adult inpatients in an Australian tertiary hospital. The first model involved the vaccination of older adult inpatients by medical officers after receiving academic detailing and weekly reminders to vaccinate (Stage 1). The second model utilised a dedicated part-time vaccine prescriber (Stage 2).

## Methods

### Study design and participants

We undertook a prospective, pragmatic evaluation with two sequential stages at a single, tertiary referral hospital in Sydney, Australia (The Prince of Wales Hospital). The evaluation ran for 12 weeks during late winter and spring (August–November 2024), a period selected to prioritise catch-up vaccination among patients who had not received seasonal respiratory immunisations. The evaluation focused on vaccines/boosters for respiratory diseases, including influenza, SARS-CoV-2 and conjugate pneumococcal vaccines. These vaccines were selected because they address a substantial burden of preventable severe disease in older adults and can be administered as single-dose vaccines during admission, avoiding reliance on post-discharge follow-up required for multi-dose schedules. Other vaccines of importance for preventing severe disease in older adults were not included. For example, RSV vaccination was not included under the Australian NIP during the evaluation period. In addition, shingles vaccination was excluded because it requires a multi-dose schedule with completion typically occurring after discharge.

Consecutive, non-elective, inpatients aged ≥ 65 years, who were admitted under medical departments (cardiology, geriatrics, geriatric rehabilitation, respiratory and infectious diseases), were screened against Australian NIP criteria for eligibility for influenza, SARS-CoV-2 and conjugate pneumococcal vaccines. Eligibility for vaccination was determined by reviewing patients’ electronic medical records (EMR) which connects to the Australian Immunisation Register (AIR; a national register of publicly funded immunisations). Patients were excluded from the evaluation if they were ineligible for all three vaccines, had been diagnosed with influenza, SARS-CoV-2 or pneumococcal disease during admission (as it is not standard of care to vaccinate inpatients with active disease), had been transitioned to end-of-life care, or had died during admission.

### Stage 1 (weeks 1–6): medical officer-led model supported by academic detailing and regular reminders to vaccinate

In Stage 1, medical officers in participating departments received a brief email outlining the rationale and workflow for inpatient vaccination (see Supplementary Material 1). The email was sent on behalf of the project team by the senior infectious diseases consultant, and the project team provided further information at ward meetings through interactive discussions. Email and ward meeting briefings are routine processes for communicating clinical practice recommendations at the hospital where the evaluation was completed. Medical officers were required to determine vaccination eligibility by reviewing the EMR. When the AIR was unavailable, or incomplete, medical officers were advised to confirm vaccination history directly with patients and/or their general practitioners. The medical officers involved in the evaluation were postgraduate adult internal medicine trainees, responsible for inpatient care under consultant specialist supervision.

Medical officers obtained verbal consent from patients, or substitute decision-makers, and prescribed vaccines in the EMR. Influenza (adjuvanted quadrivalent; Fluad Quad) and pneumococcal (13-valent pneumococcal conjugate vaccine (13vPCV); Prevenar 13) vaccines were dispensed by the hospital pharmacy and administered by nursing staff. SARS-CoV-2 (Comirnaty) vaccination required a separate booking with a dedicated nursing team that is responsible for the administration of this vaccine with the health network. This team was available Monday to Thursday. The timing of vaccination, and assessment of contraindications, was left to the discretion of the treating team. Medical officers were reminded to vaccinate and offered technical support through weekly phone calls by the project team. During these calls, feedback was also collected about the barriers encountered when vaccinations were not administered. Medical officers were collectively provided with 60 feedback opportunities, and more than one barrier per feedback opportunity could be provided. Barriers to vaccination were coded thematically. The completion of vaccinations was confirmed within the EMR.

### Stage 2 (weeks 7–12): introduction of part-time vaccine prescriber model

During Stage 2, a part-time vaccine prescriber from the project team proactively screened inpatients admitted under the cardiology, respiratory, geriatrics and geriatric rehabilitation departments. To reduce potential bias, the infectious diseases department was not included in Stage 2, as the dedicated prescriber was a member of the department. If a patient was eligible, the prescriber obtained consent from patients, or guardians, and the treating team, and prescribed vaccines in the EMR. The prescriber allocated approximately one-hour per weekday for immunisation activities. When a patient encounter did not result in vaccination, the primary barrier was recorded using a structured list. While academic detailing and weekly reminder calls to medical officers were discontinued during Stage 2, all inpatient vaccinations in departments that had participated in Stage 1 were recorded over the full duration of the evaluation. The evaluation was designed to coincide with the duration of a full rotation of medical officers within the hospital so that the enduring impact of the academic detailing could be observed during Stage 2. As with Stage 1, the completion of vaccinations was confirmed by review of vaccine administration recording in the EMR.

### Outcomes and definitions

The unit of analysis for the evaluation was a ‘vaccination opportunity’. A vaccination opportunity was defined as each potential vaccination an inpatient included in the evaluation was eligible for. Each patient was potentially eligible for up to three vaccination opportunities, influenza, SARS-CoV-2, or conjugate pneumococcal vaccine.

The pre-specified primary outcome in the evaluation was the proportion of vaccinations administered during Stage 1 versus Stage 2. Secondary outcomes included vaccine uptake per vaccine type, uptake per medical department, and barriers to vaccination.

### Data completeness

Potential misclassification bias arose from incomplete vaccination records. At least one component of vaccination history was incomplete for 257 patients (39.6%); the vaccination record was either not visible on AIR, or not documented in the EMR. These patients were still included in the evaluation as it was expected that medical officers would verify their vaccination status directly with the patient and/or with the patient’s GP as a part of routine care. This approach may have misclassified some already-vaccinated patients as eligible and not vaccinated, potentially biasing uptake downwards.

### Statistical analysis

Primary and secondary outcomes were compared using 2 × 2 tables, with odds ratios (ORs) and 95% confidence intervals. Two-sided Fisher’s exact tests were used for the primary comparison only. A generalised estimating equation (GEE) logistic model with an exchangeable working correlation, and robust standard errors, was performed to account for within-patient clustering. No formal power calculation was performed because of the predefined implementation period, with sample size determined by the number of eligible inpatients admitted during the Stage 1 and Stage 2 time windows. Secondary analyses were prespecified as exploratory and hypothesis-generating and are interpreted descriptively. Sensitivity analyses were performed to test the potential influence of (i) the higher vaccination performance of the infectious disease department, (ii) shifting the unit of analysis to per patient, rather than per vaccination, and (iii) excluding patients with incomplete vaccination records. Baseline comparability was assessed using standardised mean differences (SMDs). Analyses were conducted in R version 4.5.2.

### Ethical considerations

The Prince of Wales Hospital Human Research Ethics Committee granted a waiver of consent for the project as it was determined to be a service-evaluation study (reference number: QAQI/11JUNE2024/1). All vaccines were administered as standard of care under the Australian NIP for adults aged ≥ 65 years. Data were extracted from routine clinical records and managed in accordance with institutional policies. The evaluation was conducted in accordance with the principles of the Declaration of Helsinki.

## Results

### Baseline characteristics

As shown in Table 1, the baseline characteristics of inpatients were broadly similar between Stage 1 and Stage 2. There was, however, a small difference in the proportion admitted under the respiratory department.

**Table 1:** Patient characteristics

Characteristic	Overall	Stage 1	Stage 2	SMD
N	649	324	325	
Age, years	81.8 ± 8.0	82.2 ± 7.7	81.5 ± 8.2	-0.09
Length of stay, days (median [IQR])	6.0 (3.0–11.0)	6.0 (3.0–12.0)	6.0 (3.0–11.0)	-0.03
Female — n (%)	319 (49.2%)	160 (49.4%)	159 (48.9%)	-0.01
Eligible: Influenza — n (%)	344 (53.0%)	177 (54.6%)	167 (51.4%)	-0.07
Eligible: SARS-CoV-2 — n (%)	566 (87.2%)	286 (88.3%)	280 (86.2%)	-0.06
Eligible: Pneumococcal (conjugate) — n (%)	500 (77.0%)	247 (76.2%)	253 (77.8%)	0.04
Department: Cardiology — n (%)	218 (33.6%)	110 (34.0%)	108 (33.2%)	-0.02
Department: Geriatric Rehabilitation — n (%)	33 (5.1%)	20 (6.2%)	13 (4.0%)	-0.10
Department: Geriatrics — n (%)	240 (37.0%)	122 (37.7%)	118 (36.3%)	-0.03
Department: Infectious Diseases — n (%)	31 (4.8%)	19 (5.9%)	12 (3.7%)	-0.10
Department: Respiratory — n (%)	127 (19.6%)	53 (16.4%)	74 (22.8%)	0.16

Abbreviations: SMD, standardised mean difference; IQR, Interquartile Range

**Table 2:** Vaccination uptake in Stage 1 vs. Stage 2 by vaccine and department

		Vaccination Uptake		OR (Stage 2 vs. 1)	95% CI
	Subgroup	Stage 1 (n/N)	Stage 2 (n/N)		
By vaccine	Overall	19/710 (2.7%)	54/700 (7.7%)	3.04	1.75–5.49
	Influenza	10/177 (5.6%)	12/167 (7.2%)	1.29	0.50–3.44
	SARS-CoV-2	1/286 (0.3%)	12/280 (4.3%)	12.76	1.86–546.03
	Pneumococcal	8/247 (3.2%)	30/253 (11.9%)	4.02	1.75–10.34
By department	Overall	19/710 (2.7%)	54/700 (7.7%)	3.04	1.75–5.49
	Cardiology	0/243 (0%)	3/236 (1.3%)	7.30	0.43–Inf
	Geriatrics	5/274 (1.8%)	24/248 (9.7%)	5.76	2.10–19.60
	Geriatric Rehab	2/47 (4.3%)	8/31 (25.8%)	7.83	1.37–79.27
	Respiratory	1/105 (1%)	7/156 (4.5%)	4.89	0.61–222.07
	Infectious Diseases	11/41 (26.8%)	12/29 (41.4%)	1.93	0.62–5.97

Notes: ORs use a 0.5 continuity correction when any cell is zero

### Participant flow

**Fig. 1:**
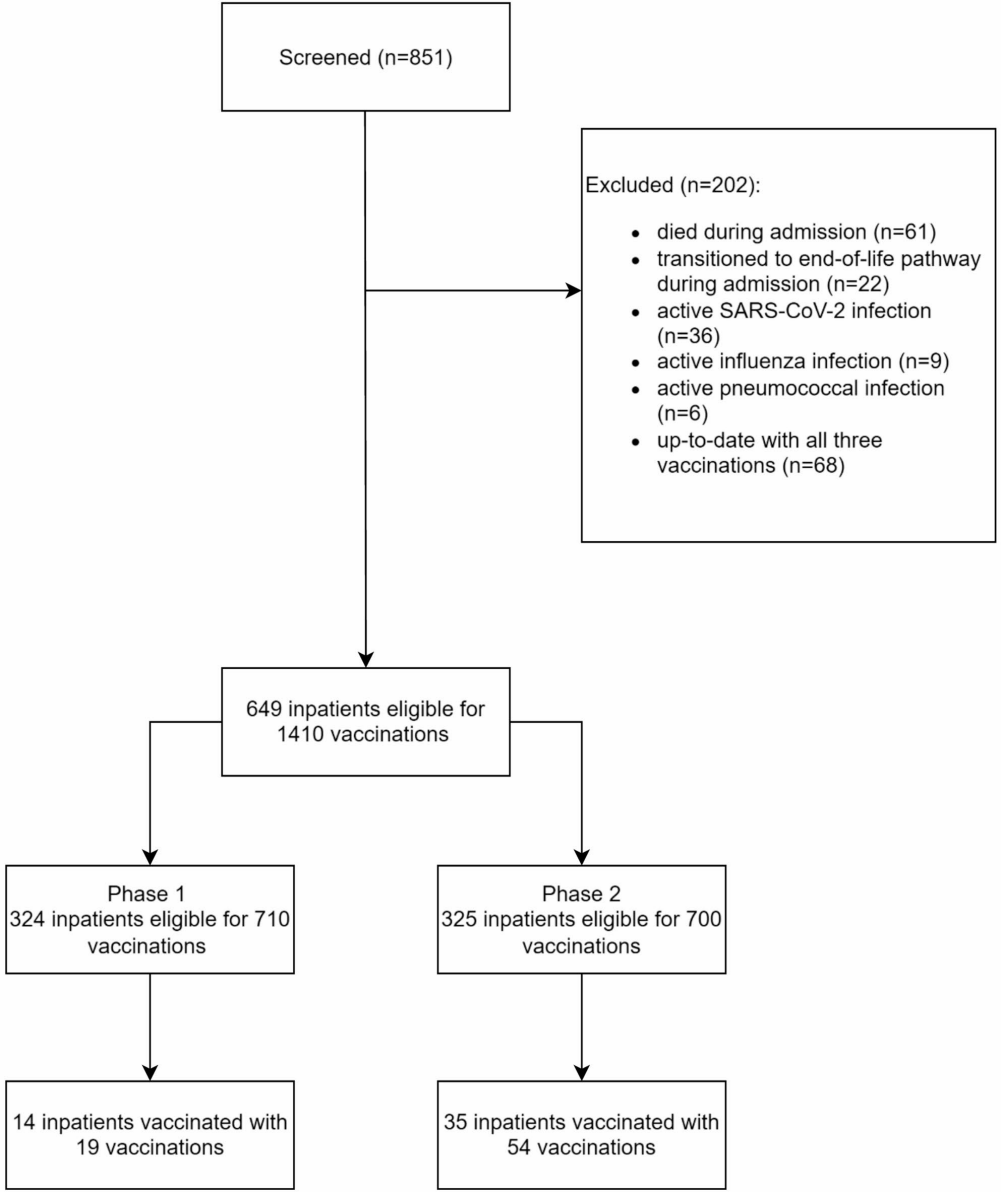
Study flowchart

During the full 12-week duration of the evaluation, 851 inpatients aged ≥ 65 years were screened. There was a total of 1,410 vaccination opportunities for the 649 inpatients who met the inclusion criteria. Of the 202 patients who were ineligible to participate, 68 were up to date with their vaccinations, 36 had active SARS-CoV-2 infection, 9 had active influenza infection, 6 had active pneumococcal disease, 22 had transitioned to an end-of-life pathway during admission, and 61 had died during admission. The size of the two patient groups in Stage 1 (*n* = 324) and Stage 2 (*n* = 325) was almost the same. Figure 1 shows the study flow.

**Fig. 2:**
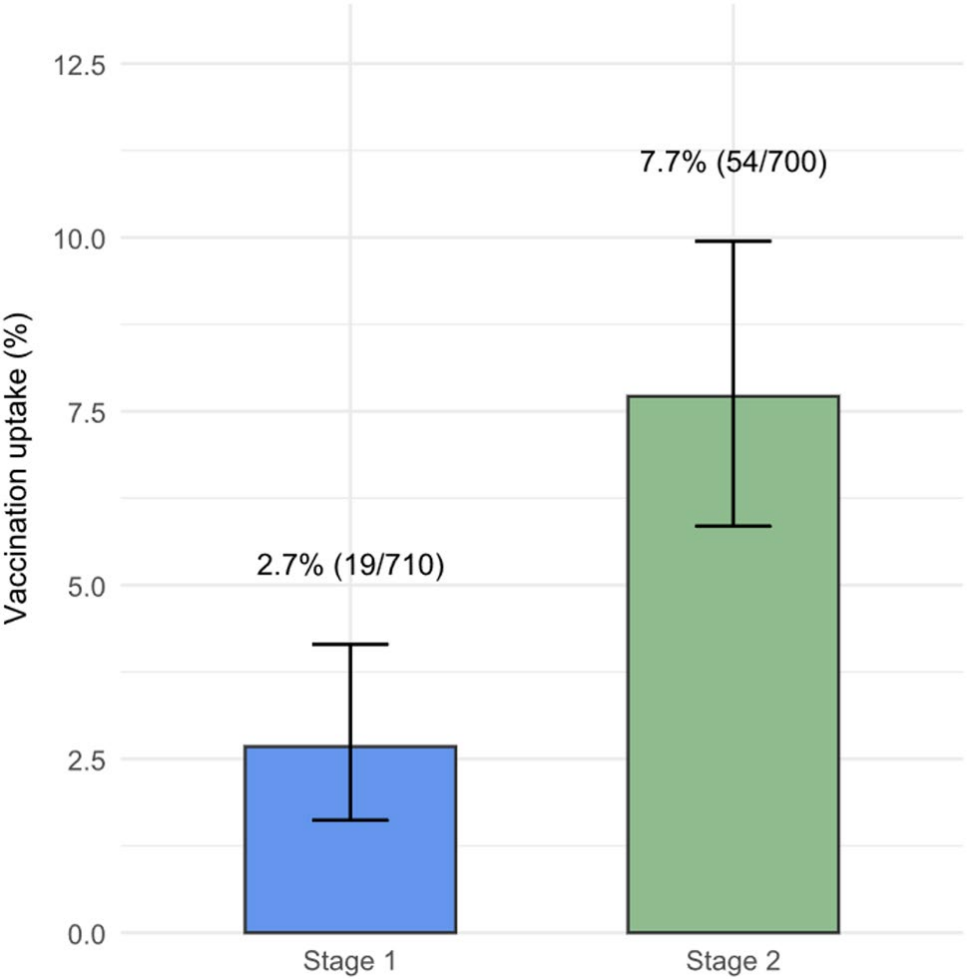
Total vaccination uptake: Stage 1 vs. Stage 2

**Fig. 3:**
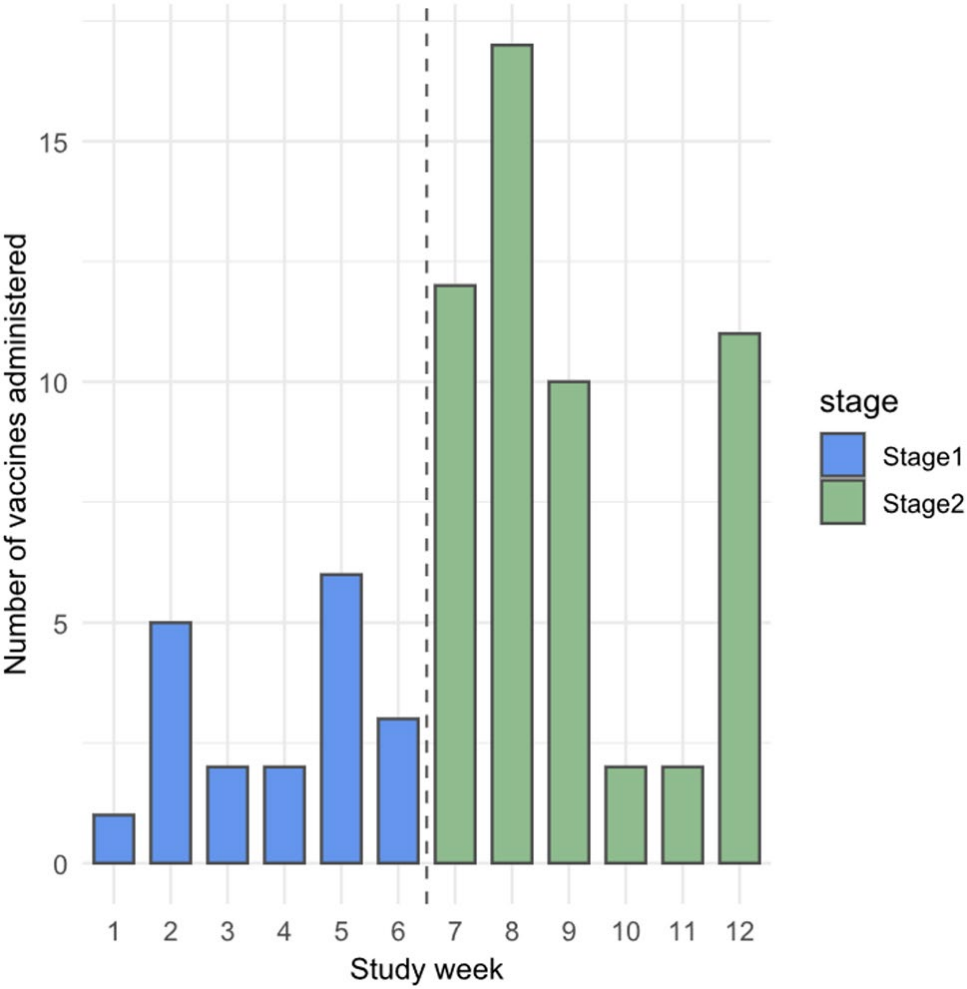
Weekly vaccination uptake over the evaluation period, by stage

**Fig. 4:**
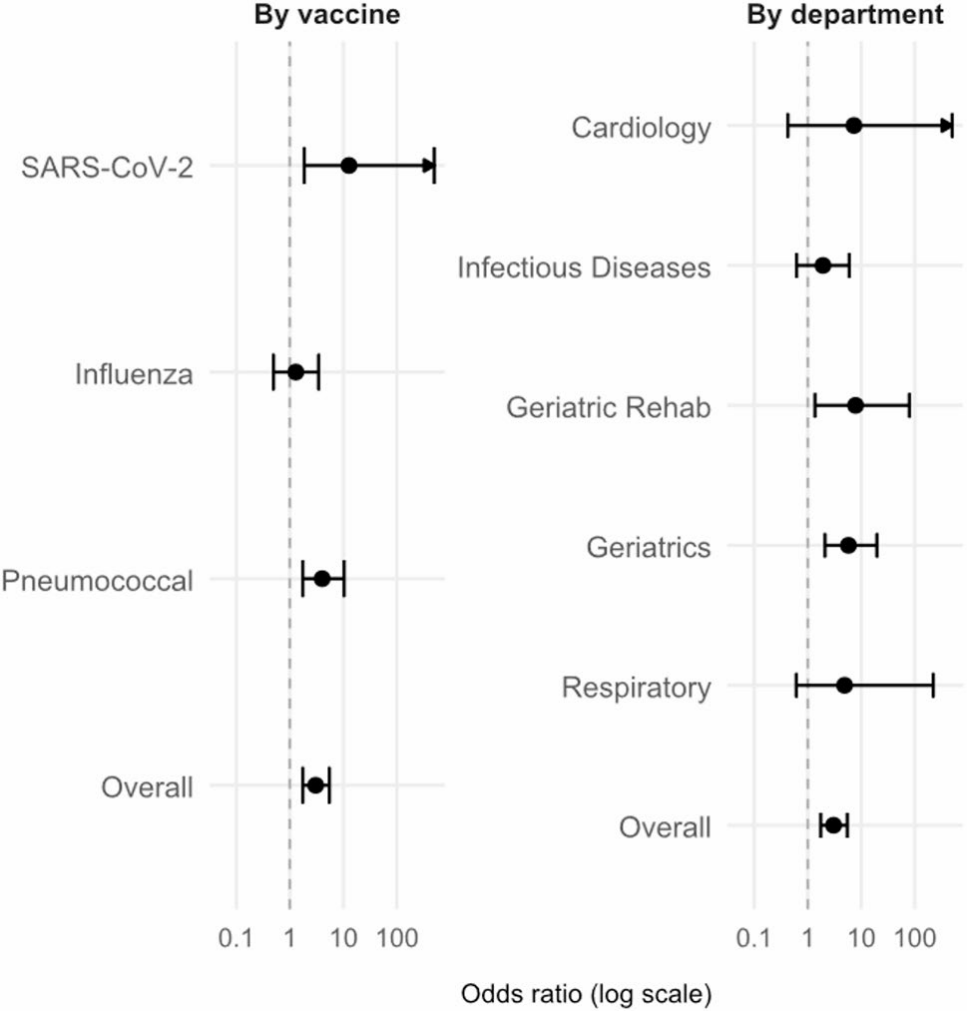
Inpatient Vaccination Uptake: Stage 2 vs. Stage 1 by vaccine and by department. Numeric values for the figure are provided in Table2

**Fig. 5:**
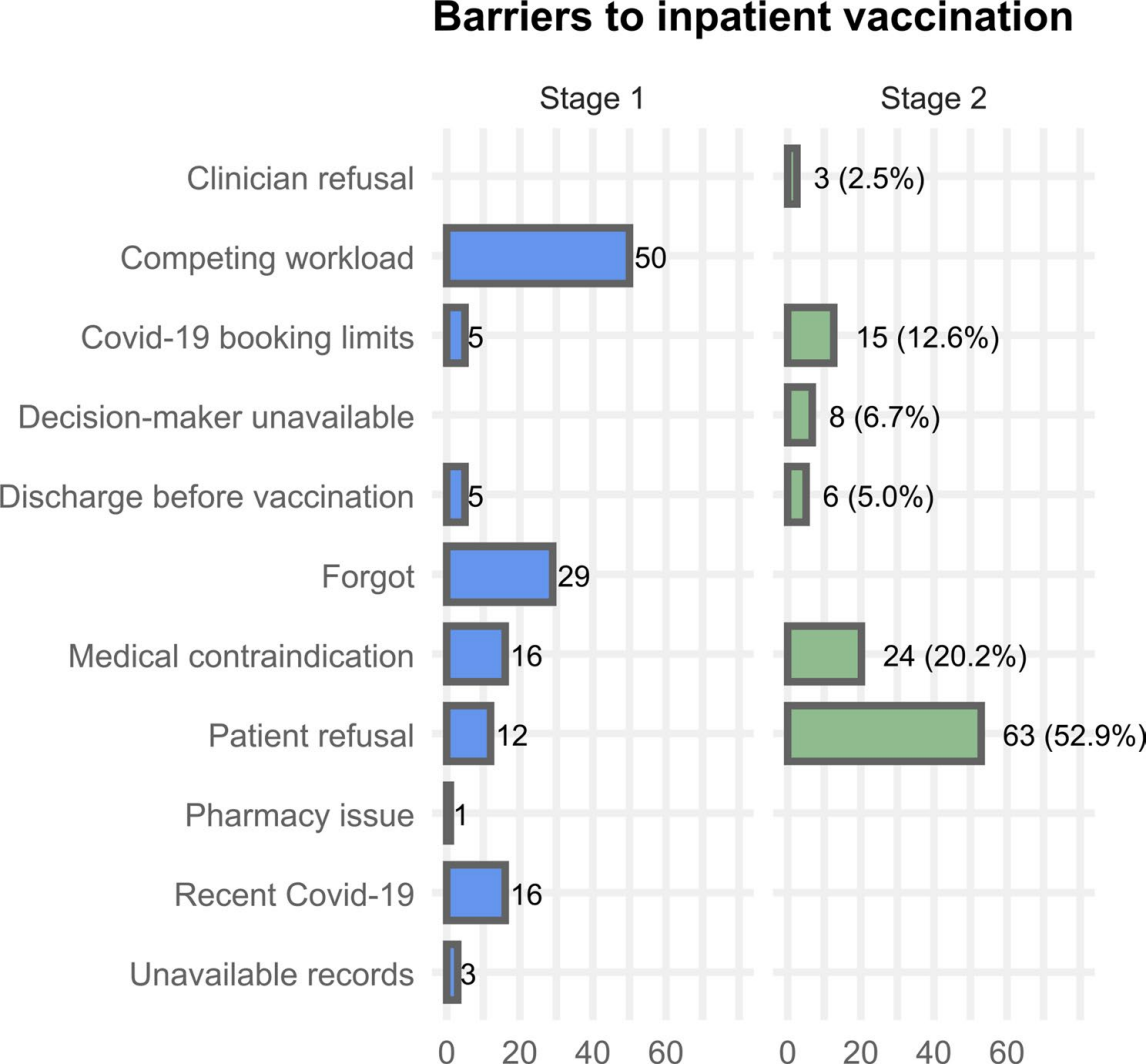
Barriers to vaccination. Stage 1 panel includes counts only (no denominator). Stage 2 panel includes proportions of encounters (*n* = 119)

#### Primary outcome

Overall, inpatient vaccination increased from 19/710 (2.7%) during Stage 1, to 54/700 (7.7%) during Stage 2 (OR 3.04, 95% CI 1.75–5.49; p < 0.001) (see Fig. 2). Of the 54 vaccines administered in Stage 2, 39 (72.2%) were delivered by the dedicated vaccine prescriber (Model 2), and 15 (27.8%) were administered by medical officers (Model 1), mostly in the infectious diseases department (12/15, 80%). The cluster-adjusted GEE gave a similar Stage 2 effect (OR 2.96, 95% CI 1.53–5.73; p = 0.001). Vaccination uptake by week is shown in Fig. 3.

#### Secondary outcomes

When data were analysed by vaccine type, uptake improved for SARS-CoV-2 (12/280 vs. 1/286; OR 12.7, 95% CI 1.86–546.03) and pneumococcal vaccination (30/253 vs. 8/247; OR 4.00, 95% CI 1.75–10.34), but not for influenza vaccination (12/167 vs. 10/177; OR 1.29, 95% CI 0.50–3.44). See Table 2  and Fig. 4.

#### Sensitivity analyses

After excluding the infectious diseases department, vaccination uptake increased from 8/669 (1.2%) in Stage 1, to 42/671 (6.3%) in Stage 2 (OR 5.52, 95% CI 2.57–11.84), suggesting the observed increase was not limited to the infectious diseases department. 

When analysed per patient, rather than per vaccine opportunity, 14/324 (4.3%) patients in Stage 1, and 35/325 (10.8%) in Stage 2 received, at least one vaccine (OR 2.67, 95% CI 1.39–5.15). Excluding the infectious diseases department, 6/305 (2.0%) patients in Stage 1, and 30/313 (9.6%) patients in Stage 2, were vaccinated (OR 5.27, 95% CI 2.12–15.73). These results were consistent with the primary findings when assessed at the patient level.

After excluding patients with incomplete vaccination records, vaccination uptake increased from 19/269 (7.1%) in Stage 1 to 54/304 (17.8%) in Stage 2 (OR 2.84, 95% CI 1.60–5.22). Incomplete records occurred at similar frequencies across stages (Stage 1: 130 patients; Stage 2: 127 patients).

#### Barriers to inpatient vaccination

Barriers to inpatient vaccination in both stages are summarised in Figure 5. During Stage 1, the most commonly identified barriers to vaccinating older adult inpatients were competing priorities and workload (n=50), forgetting to vaccinate (n=28), patients being too unwell (n=16), patients having recently had SARS-CoV-2 infection (n=16), and patient refusal (n=12). Less common barriers included discharge before vaccination (n=5), difficulties organising SARS-CoV-2 vaccination appointments (n=5), unavailable records (n=3), and a pharmacy dispensing issue (n=1).

The most common barriers experienced by the dedicated vaccine prescriber during Stage 2 included patient refusal (63/119, 52.9%) and medical contraindications (24/119, 20.2%). The reasons provided by patients for vaccine refusal included concerns about side-effects (6/63, 9.5%), a preference to vaccinate with their GP (5/63, 7.9%), reluctance to receive multiple vaccines concurrently (3/63, 4.8%), the vaccines being outside their ceiling of care (3/63, 4.8%), and vaccine scepticism (2/63, 3.2%). Less common barriers experienced by the dedicated vaccine prescriber included difficulties organising SARS-CoV-2 vaccination appointments (15/119, 12.6%), difficulties contacting the patient’s decision-maker (8/119, 6.7%), the patient being discharged before vaccination (6/119, 5.0%), and the treating team’s refusal to vaccinate (3/119, 2.5%). 

### Discussion

This evaluation found that an inpatient vaccination model utilising a part-time vaccine prescriber (1 h per weekday) increased older adult inpatient vaccination rates approximately threefold compared to a model involving academic detailing and vaccination reminders sent to medical officers. The difference in effectiveness of the two models appears to be explained by the barriers to vaccination reported by the medical officers and dedicated vaccine prescriber. During Stage 1, the most common barriers identified by medical officers were competing priorities and forgetting. These barriers were removed through the introduction of a dedicated vaccine prescriber during Stage 2. The remaining barriers were mostly patient-related, such as vaccine refusal, medical contraindication, discomfort with the setting, and vaccine scepticism. This indicates that workforce constraints, not education and reminders, is the primary modifiable barrier to improve inpatient vaccination rates

The data showed that infectious diseases medical officers were more likely to be influenced by academic detailing and vaccination reminders than medical officers from other participating departments. This is likely due to stronger staff engagement and ownership of vaccination tasks by clinical staff working within an infectious diseases department. Achieving such conditions among other medical departments might be possible, however, it has been shown that accomplishing cultural change within hospital settings can be difficult [17]. By contrast, introducing a dedicated vaccine prescriber provides an immediate and scalable way to increase uptake in other departments

### Comparison with prior studies

Our findings are consistent with previous studies evaluating opportunistic vaccination of adult inpatients; however, the research conducted on this topic is limited. Few studies have specifically assessed dedicated vaccine prescriber/vaccinator, or staff education, interventions within hospital settings. In Australia, Davies et al. (2025) evaluated a dedicated vaccinator program delivered by Authorised Nurse Immunisers (ANIs) that achieved high uptake (398/849; 46.9%) across three hospitals, outpatient clinics and community centres over ten weeks [16]. Davies’ et al. (2025) higher absolute vaccination rate compared to our dedicated vaccine prescriber model (7.7%) likely reflects their use of ANIs who completed all vaccination steps, including confirming eligibility, consenting, and administration. This approach effectively removed the opportunity for downstream barriers that were experienced within our evaluation. Another reason why our vaccination rates were lower may be Davies’ et al. (2025) inclusion of a lower-acuity patient population (including outpatients) and exclusion of any patients with medical contraindications. Their inpatient participants had a median hospital stay of 26 days. In contrast, our evaluation focused on a high-risk group of inpatients for whom hospitalisation may represent the only opportunity for vaccination. We included acutely unwell medical inpatients, with minimal exclusions, and a median hospital stay of just 6 days. It is also likely that the vaccinators in the program evaluated by Davies et al. (2025) had greater capacity than in our intervention, with multiple ANIs working across several sites and days per week (however, total vaccinator hours were not reported in their paper). Despite contextual differences, both studies suggest that utilising dedicated vaccination staff is an effective approach to increasing hospital-based immunisation

Similar to our evaluation, previous research has also shown that education-focused strategies only produce modest gains in opportunistic vaccination in hospital settings. De Guzman et al. (2022) reported a 4.7% increase in inpatient COVID-19 vaccination following a 3-month single-service quality improvement intervention targeting staff and patient education [18]. Murray et al. (2020) and Rivière et al. (2023) assessed clinician- and patient-education interventions to increase vaccination uptakes in hospital specialist outpatient clinics, and found no significant change [19, 20]. Similarly, a systematic review by Akther et al. (2025) examining 42 studies on healthcare workers’ pneumococcal vaccination practices, found that knowledge and motivation were generally high, yet uptake was constrained by time pressure, logistics, and limited organisational support [21]. Building on this literature, our evaluation adds pragmatic comparative data by examining two sequential inpatient vaccination delivery models within the same hospital. Collectively, these findings show that education alone is not sufficient to markedly increase inpatient vaccination as workload and system-level barriers remain. Interventions that add dedicated vaccination capacity appear to achieve greater and more sustainable improvements

### Practical implications

Allocating limited time for vaccination activities within existing clinical workflows offers a pragmatic way to improve vaccination among high-risk inpatients. Academic detailing and reminders remain valuable for increasing awareness among medical staff, however, they appear insufficient to overcome workload constraints by themselves. The economic impact of an inpatient vaccination program was not assessed in this evaluation. However, prior Australian work evaluating inpatient influenza vaccination has reported potential cost savings for the healthcare system of approximately AUD $306 per vaccinated patient, per year, reflecting the established efficacy of vaccination in reducing hospital admissions [22]. Future studies should formally evaluate resource requirements and cost implications alongside effectiveness. In our evaluation, allocating approximately one hour of vaccine prescriber time per weekday in a high-acuity setting achieved a marked increase in inpatient vaccination uptake. Further increase in inpatient vaccination rates will likely require improved patient education and clearer clinical guidance on vaccination timing and eligibility in medically unwell inpatients

While the evaluation provided important findings on how to improve opportunistic vaccination for older adult inpatients, several potential limitations should be acknowledged. The evaluation did not assess baseline inpatient immunisation rates prior to the commencement of the vaccination models; these were assumed to be negligible, given our knowledge of local practices and the findings of research conducted in similar settings [23]. It is also possible that initiating the vaccination models in sequence, rather than in parallel, introduced temporal and/or seasonal bias (such as, but not limited to, staffing and workload changes, other vaccination campaigns or messaging). Week-by-week data showed uptake increased soon after the dedicated prescriber commenced; however, temporal confounding cannot be excluded. To our knowledge, there were no other hospital vaccination campaigns, or major workload changes, during the evaluation period. Also, the medical offers on participating wards remained the same throughout the evaluation.

The modest change in influenza vaccination uptake between the two stages is most likely explained by the smaller number of influenza-eligible patients as the influenza season (June to September in the southern hemisphere [24]) had finished. As this evaluation was conducted in a tertiary hospital in a high-income country, caution also needs to be taken when generalising the findings to other settings, especially those with fewer resources. More detailed patient-level data (including ethnicity and baseline comorbidities) would also be valuable to assess subgroup differences in eligibility and uptake, and to support comparability between stages. Finally, the evaluation did not assess cost-effectiveness or long-term sustainability of the two vaccination models; this should be investigated in future research.

### Conclusion

In this prospective evaluation of two models for opportunistic immunisation of older adult inpatients, a part-time vaccine prescriber model achieved higher vaccination uptake than academic detailing and reminder-based support for medical officers. Introducing a dedicated vaccine prescriber working approximately one hour per weekday was associated with an approximate threefold increase in inpatient vaccination uptake, with notable gains for SARS-CoV-2 and pneumococcal vaccines in a high-risk population with short lengths of stay.

The findings suggest that limited improvements in opportunistic inpatient vaccination are unlikely to be resolved by education and reminders alone. Instead, constrained workforce capacity and competing clinical priorities appear to be the key modifiable barriers. Allocating time for vaccination activities within existing hospital workflows may offer a pragmatic strategy to improve vaccination coverage for older adults and may be particularly valuable in tertiary settings where hospitalisation represents a critical opportunity to vaccinate patients who may not routinely access primary care.

Given the single-centre design and the relatively low absolute uptake achieved, further research is needed to examine the cost-effectiveness, scalability, and long-term sustainability of dedicated prescriber and vaccinator models, including in different hospital types and health-system contexts. Future studies should also explore how best to address patient-level barriers identified in this evaluation, such as refusal, concerns about side-effects, and uncertainty about vaccination during acute illness.

## Supplementary Information

Below is the link to the electronic supplementary material.

Supplementary Material 1

## Data Availability

The datasets generated and/or analysed during the current evaluation are available from the corresponding author on reasonable request.

## Abbreviations

13vPCV: 13-valent pneumococcal conjugate vaccine
AIR: Australian Immunisation Register
ANI: Authorised Nurse Immuniser
AUD: Australian dollars
CI: Confidence interval
EMR: Electronic medical record
GEE: Generalised estimating equation
GP: General practitioner
ID: Infectious Diseases
IQR: Interquartile range
NIP: National Immunisation Program
OR: Odds ratio
SARS-CoV-2: Severe acute respiratory syndrome coronavirus 2
SMD: Standardised mean difference
