# Supplementary material for: Hospital-based vaccination of older adult inpatients: comparison of two delivery models for influenza, COVID-19 and pneumococcal vaccines
Source: BMC Infect Dis. 2026 Mar 2;26:729. doi: 10.1186/s12879-026-12970-y (PMC13059362; doi:10.1186/s12879-026-12970-y)
Supplement: Supplementary file 1 — Supplementary Material 1 [file 12879_2026_12970_MOESM1_ESM.pdf]

Dear Consultants and Registrars,

In 2023 an audit found that the rates of appropriate immunisation against influenza, pneumococcus and COVID-19 for people aged over 65 years admitted to Prince of Wales Hospital were only 61%, 21% and 21%. We **seek your help** to improve these rates by immunising eligible people admitted to hospital. This should reduce length of stay of those infected and reduce readmissions.

Check eligibility
Patients are eligible for free immunisation based on age as outlined in the flowchart below:

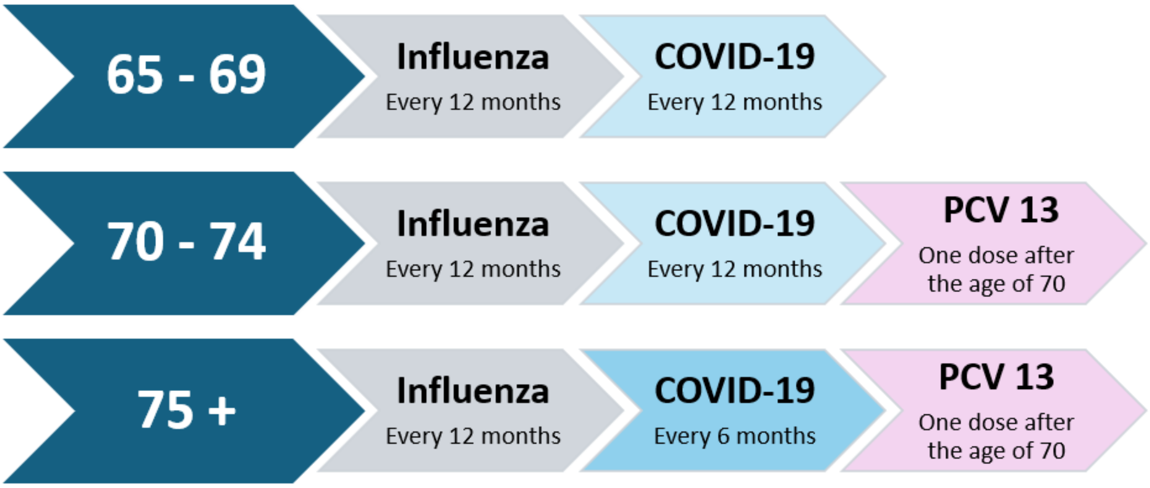

There are different recommendations for Aboriginal and Torres Strait Islanders, people with comorbidities and people with immunodeficiency. See The Australian Immunisation Handbook (health.gov.au)

You can check the patient's vaccination record on HealtheNet via Powerchart.

Timing of the Vaccine
Administer the vaccine when the patient is medically stable. Patients recovering from acute infections can be vaccinated once they have been afebrile for 48 hours. The timing of vaccination should be at the medical team's discretion.

Concurrent Administration of Multiple Vaccines
COVID-19, influenza, and Prevenar 13 (pneumococcal) vaccines can be administered simultaneously.

Consent
Obtain verbal consent after discussing risks and benefits with the patient or a substitute decision maker.

Chart the Vaccine on EMR

- To give Influenza vaccine, chart inactivated quadrivalent Fluad Quad IM on EMR and notify the ward nursing staff to administer it.
- To give pneumococcal vaccine (Prevenar-13), chart 13-valent pneumococcal conjugate vaccine IM on EMR and notify the ward nursing staff to administer it.
- To give COVID-19 vaccine, complete a form (see attachment) and email to Staff Health ([redacted]) to organise a vaccination booking. Note that

the Staff Health Service is located at St George Hospital and is not available at POWH on Fridays or weekends. You then need to chart Comirnaty OmicronXBB.1.5 COVID-19 Vaccine on EMR.

Vaccine Record
Vaccinations administered via EMR are automatically recorded with the Australian Immunisation Register. It is recommended to include vaccination details in the discharge summary.

Contact
Dr [redacted] (pager [redacted]) will be in touch with medical teams weekly to assist with problem solving.

Best regards,
